# Comparison of Transperitoneal Versus Retroperitoneal Laparoscopic Pyeloplasty in Terms of Efficacy and Safety: A Systematic Review and Meta-Analysis of Pediatric Patients

**DOI:** 10.7759/cureus.112055

**Published:** 2026-07-04

**Authors:** Tri Endah Suprabawati, Randy Fauzan, Irfan Wahyudi, Arry Rodjani, Gerhard Reinaldi Situmorang, Putu Angga Risky Raharja

**Affiliations:** 1 Department of Urology, Budi Asih Regional General Hospital, Jakarta, IDN; 2 Department of Urology, Universitas Indonesia, Jakarta, IDN; 3 Department of Urology, Rumah Sakit Umum Pusat Nasional Dr. Cipto Mangunkusumo, Jakarta, IDN

**Keywords:** laparoscopic pyeloplasty, meta-analysis, pediatrics, retroperitoneal, transperitoneal, ureteropelvic junction obstruction

## Abstract

Laparoscopic pyeloplasty is currently recognized as the standard of care for pediatric ureteropelvic junction obstruction. However, the choice between laparoscopic transperitoneal (LT) and laparoscopic retroperitoneal (LR) approaches remains a subject of ongoing debate, as existing literature presents conflicting reports regarding operative efficiency and hospitalization duration. To compare the efficacy and safety of LT versus LR in pediatric patients and investigate sources of heterogeneity using meta-regression, a systematic review was performed following the Preferred Reporting Items for Systematic Reviews and Meta-Analyses (PRISMA) guidelines and searching PubMed, Cochrane Library, and Google Scholar for all records published up to December 19, 2025. Data were synthesized using a random-effects meta-analysis. Meta-regression and restricted cubic spline analyses were employed to assess the influence of study design, country, and patient age on heterogeneity (I2>90%). Six comparative studies (one randomized controlled trial and five retrospective studies) were included. Pooled analysis showed no significant differences in success rates (risk ratio = 1.00; 95% confidence interval = 0.96-1.04), complications, or conversion rates. However, meta-regression revealed that study design significantly influenced operative time (p=0.035), with retrospective studies exaggerating time differences compared to randomized trials. Additionally, country of origin was the primary driver of hospital stay variation (p=0.013; R2 of approximately 70%). Exploratory restricted cubic spline analysis suggested a possible age-related inflection point for conversion risk at approximately 2.06 years; however, this finding should be considered hypothesis-generating because of the limited number of included studies. LT and LR demonstrate comparable safety and efficacy. Variability in operative time and hospitalization is largely driven by study design biases and geographic protocols rather than intrinsic technical differences. While both techniques are effective, anatomical constraints in infants under two years warrant careful consideration.

## Introduction and background

Congenital ureteropelvic junction obstruction (UPJO) is the most common cause of pathological hydronephrosis in children and frequently requires surgical correction to preserve renal function and prevent long-term renal damage [1,2]. Over the past decades, minimally invasive pyeloplasty has progressively replaced conventional open surgery because it is associated with lower postoperative morbidity, improved cosmetic outcomes, and faster postoperative recovery while maintaining comparable surgical success [3,4].

UPJO is characterized by intrinsic narrowing or functional aperistalsis at the junction of the renal pelvis and proximal ureter, resulting in impaired urinary drainage and progressive dilatation of the pelvicalyceal system. Persistent obstruction may ultimately lead to irreversible nephron loss and progressive renal insufficiency if left untreated [1,2]. The Anderson-Hynes dismembered pyeloplasty remains the gold-standard surgical treatment regardless of whether it is performed through an open, laparoscopic, or robot-assisted approach, with reported success rates consistently exceeding 90% [3,4]. Although three previous meta-analyses have compared transperitoneal and retroperitoneal laparoscopic pyeloplasty in mixed pediatric and adult populations (Wu et al. 2012 [5], Chua et al. 2020 [6], and Ji et al. 2021 [7]), none incorporated meta-regression to investigate potential sources of heterogeneity. Furthermore, these studies did not specifically address the anatomical limitations of the pediatric retroperitoneal space, which is substantially narrower in infants and toddlers than in older children or adults, potentially increasing operative complexity and the likelihood of conversion, particularly in patients younger than two years [8].

Among minimally invasive techniques, the transperitoneal approach is widely adopted because its larger working space and familiar anatomical orientation facilitate intracorporeal suturing and shorten the technical learning curve [9,10]. Conversely, the retroperitoneal approach provides direct access to the ureteropelvic junction without entering the peritoneal cavity, theoretically reducing the risks of postoperative ileus, visceral injury, and intra-abdominal adhesion formation [11,12].

Despite these theoretical advantages, the comparative effectiveness of both approaches remains controversial. Several studies have demonstrated improved operative efficiency with the retroperitoneal approach owing to more direct anatomical access [13], whereas others have reported greater technical difficulty and higher conversion rates, especially in younger children with limited retroperitoneal working space [14]. Moreover, previous systematic reviews have largely synthesized these findings without adequately exploring the substantial clinical and methodological heterogeneity across studies. Important factors, including patient age, study design (randomized versus observational), and regional differences in institutional practice, may significantly influence treatment outcomes but have not been systematically evaluated [5,6,13].

Accordingly, the present study aims not only to provide an updated comparison of the efficacy and safety of transperitoneal versus retroperitoneal laparoscopic pyeloplasty but also to identify determinants of between-study heterogeneity through a comprehensive meta-regression analysis.

## Review

Methods

Protocol and Registration

This meta-analysis was conducted in accordance with the Preferred Reporting Items for Systematic Reviews and Meta-Analyses (PRISMA) guidelines [15] and the Cochrane Handbook for Systematic Reviews of Interventions, version 6.5 (2024) [16]. This study’s protocol has been registered in the PROSPERO database (CRD420251081336).

Search Strategy and Data Sources

A comprehensive systematic literature search was conducted across major electronic databases, including PubMed, The Cochrane Library, and Google Scholar, covering all records published up to December 19, 2025. The search strategy employed a combination of Medical Subject Headings (MeSH) terms and free-text keywords using Boolean operators to maximize sensitivity.

The specific search string utilized the following keyword combinations: (("Laparoscopic pyeloplasty" OR "Minimally Invasive Pyeloplasty" OR "Keyhole Pyeloplasty" OR "Endoscopic Pyeloplasty" OR "Laparoscopic-Assisted Pyeloplasty" OR "Robotic-Assisted Pyeloplasty") AND ("Intraperitoneal" OR "peritoneal") AND ("retroperitoneal" OR "Extraperitoneal" OR "Posterior approach" OR "Retroperitoneoscopic" OR "Dorsal approach")). Per-database search strings are fully documented in Table 1.

**Table 1:** Boolean search strategy, keywords used for each database, and the number of records retrieved.

Database	Search String (adapted)	Search	Records Retrieved
PubMed	(laparoscopic assisted surgery[MeSH Terms]) OR (Minimally Invasive Pyeloplasty[Title/Abstract] OR Keyhole Pyeloplasty[Title/Abstract] OR Endoscopic Pyeloplasty[Title/Abstract] OR Laparoscopic-Assisted Pyeloplasty[Title/Abstract] OR Robotic-Assisted Pyeloplasty[Title/Abstract])	#1	128,845
	(intraperitoneal[Title/Abstract] OR transperitoneal[Title/Abstract]) AND (peritoneal[Title/Abstract])	#2	16,114
	Extraperitoneal[Title/Abstract] OR l retroperitoneoscopic[Title/Abstract] OR Posterior approach[Title/Abstract] OR Dorsal approach[Title/Abstract]	#3	13,737
	#1 AND #2 AND #3		107
Cochrane Library (CENTRAL)	MeSH descriptor: [laparoscopy] explode all trees	#1	9,772
	(Minimally Invasive Pyeloplasty OR Keyhole Pyeloplasty OR Endoscopic Pyeloplasty OR Laparoscopic-Assisted Pyeloplasty OR Robotic-Assisted Pyeloplasty): ti,ab,kw	#2	36
	#1 OR #2	#3	9,746
	(intraperitoneal OR transperitoneal):ti,ab,kw	#4	4,396
	(Extraperitoneal OR retroperitoneoscopic OR Posterior approach OR Dorsal approach):ti,ab,kw	#5	4,355
	#3 AND #4 AND #5		32
Google Scholar	"laparoscopic pyeloplasty" AND ("transperitoneal" OR "intraperitoneal") AND ("retroperitoneal" OR "extraperitoneal") — first 200 result pages screened for relevance		890

A grey literature search was performed via Google Scholar. While maximizing inclusivity, we acknowledge the potential for selection bias due to the lack of predefined screening thresholds for this database.

Eligibility Criteria: Inclusion and Exclusion

Before starting the literature search, inclusion and exclusion criteria were established a priori to ensure the relevance of the data collected.

The inclusion criteria comprised studies that performed laparoscopic pyeloplasty, studies that directly compared transperitoneal and retroperitoneal laparoscopic approaches, studies that examined outcomes related to efficacy and/or safety and/or incidence of complications, and articles written in English. Conversely, the exclusion criteria consisted of studies that did not report complete and clear results, small-scale studies with fewer than three patients, studies that included adult patient populations (greater than 18 years), and articles that did not have access to the full text.

Study Selection and Quality Assessment

**Figure 1:**
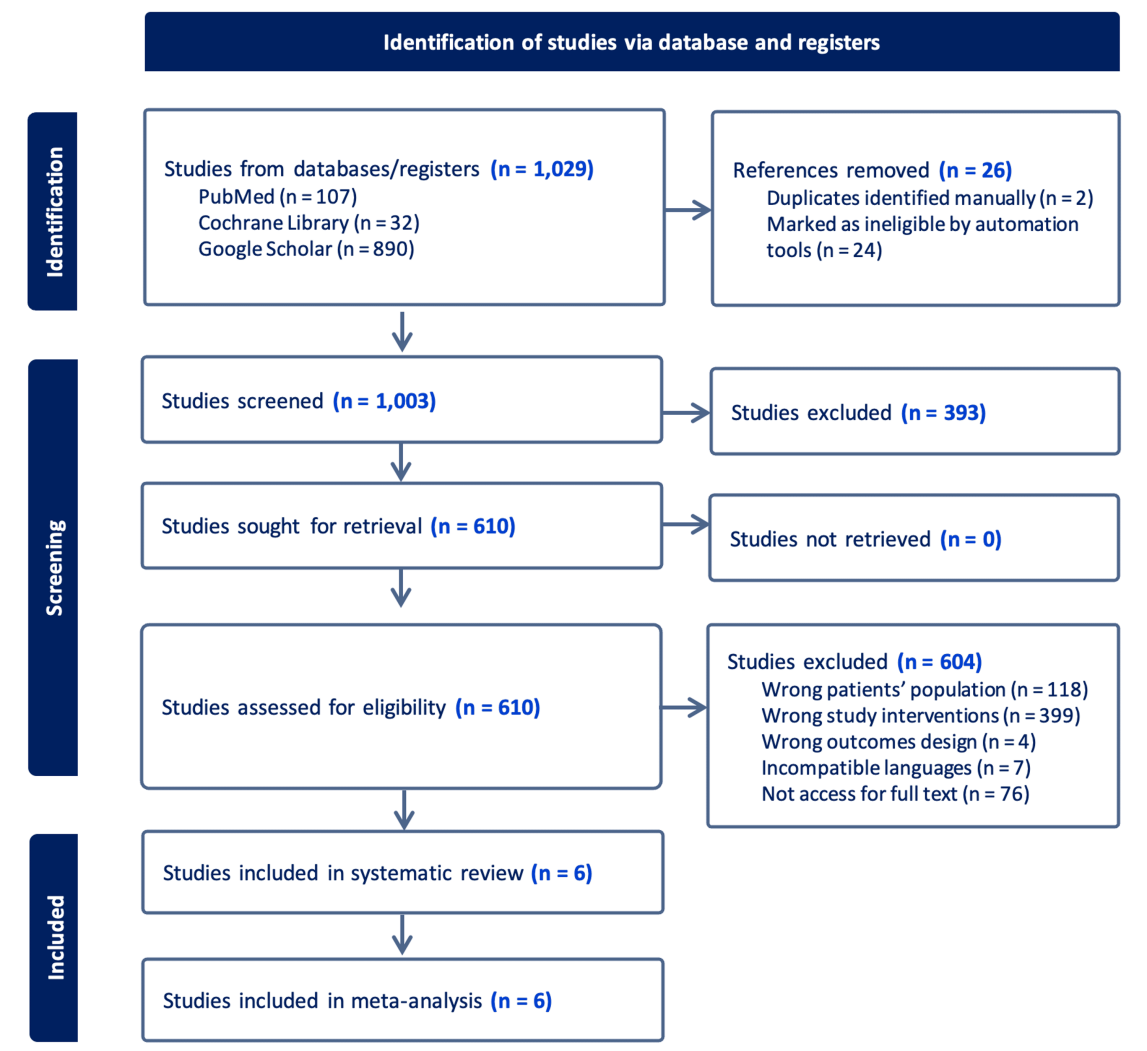
PRISMA flow diagram detailing literature identification, screening, eligibility assessment, and final study inclusion. PRISMA: Preferred Reporting Items for Systematic Reviews and Meta-Analyses

Two independent reviewers screened the studies for inclusion; disagreements were resolved by consensus. The initial systematic search across electronic databases identified a total of 1,029 records. The systematic process of selecting inclusion studies is illustrated in Figure 1. References were obtained from randomized controlled trials (RCTs), clinical trials, or cohort studies that evaluated the efficacy and safety of laparoscopic transperitoneal and retroperitoneal approaches to pyeloplasty. 

**Figure 2:**
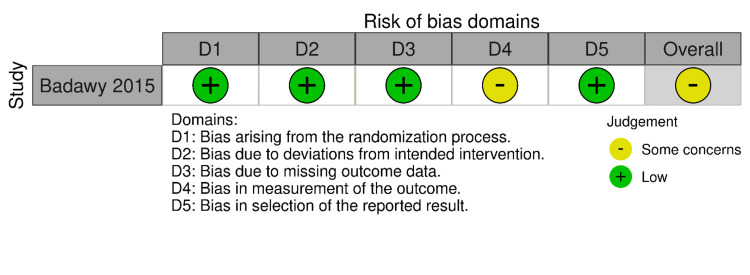
Analysis of the risk of bias using the RoB 2 tool for randomized controlled trials. RoB 2: Risk of bias 2

Journal quality and risk of bias analyses were conducted according to the research study design. For the inclusion studies with an RCT study design, the risk of bias was assessed using the Risk of Bias 2 (RoB 2) tool [17], and for studies with retrospective designs, we used the Newcastle-Ottawa Scale [18]. Prior to the quality assessment and data extraction, we established protocols for handling missing continuous data [19,20] and planned our meta-regression framework [21-23]. The retrospective cohorts subjected to this quality evaluation using the Newcastle-Ottawa Scale included the studies by Marei et al. [24], Zhang et al. [25], Liu et al. [26], and El-Gohary [27]. Data from the risk of bias assessment can be seen in Figure 2 and Table 2.

**Table 2:** Analysis of the risk of bias using the Newcastle-Ottawa Scale for retrospective design studies. Scores are presented as integers for each domain: Selection (maximum 4), Comparability (maximum 2), and Outcome (maximum 3), giving a theoretical maximum of 9. Quality grading: total score ≥7 = High methodological quality; 4–6 = Moderate quality; <4 = Low quality.

Study	Selection	Comparability	Outcomes
Marei 2025 [24]	****	*	**
Zhang 2019 [25]	****	*	***
Liu 2016 [26]	****	**	***
El-Gohary 2012 [27]	****	*	**
Canon 2007 [14]	****	*	**

Data Extraction and Management

The data extracted from the inclusion studies included the authors' names, year of publication, title, country of origin, study design, number of participants, population characteristics, and outcome parameters such as operative time, conversion from laparoscopic to open surgery, success rate, definition of success, and complications based on the Clavien-Dindo classification [28].

Data were presented as mean (SD) and numbers of events and non-events. If mean (SD) data were not available, the authors used statistical estimation using the method described by Wan et al. [19]; and also weighted mean estimation was performed, and standard error of the mean (SEM) was converted to standard deviation (SD), and missing standard deviations were imputed by borrowing SDs in accordance with the Cochrane Handbook [20]. The comprehensive data extraction framework for the included studies is summarized in Table 3.

**Table 3:** Data extraction of the included studies. NI: no information; CT: clinical trial; RCT: randomized controlled trial; LP: laparoscopic pyeloplasty; RALP: robot-assisted laparoscopic pyeloplasty; SC: single center; MC: multicenter; TC: two centers. *Data required conversion and estimation to mean (SD) using the method described by Wan et al. [19]. †Weighted mean estimation was performed. ‡Standard error of the mean (SEM) was converted to standard deviation (SD). §Missing standard deviations were imputed by borrowing SDs in accordance with the Cochrane Handbook [20].

Author, Year	Study Design	n (LR/LT)	Center	Characteristics of populations (mean (SD))							
				Age (Years)		Male /Female		Right Side/Left Side		Crossing Vessel	
				LR	LT	LR	LT	LR	LT	LR	LT
Marei 2025 [24]	Retrospective	56/40	MC	10.5 (3.25)^†^	7.9 (4.4)^†^	NI	NI	NI	NI	NI	NI
Zhang 2019 [25]	Retrospective	22/14	SC	1 (0.5)	0.95 (0.47)	10/12	NI	NI	NI	NI	NI
Liu 2016 [26]	Retrospective	451/311	SC	3.5 (6.58)^‡^	3.2 (4.06)^‡^	303/148	210/101	96/325 (30 bilateral)	71/219 (21 bilateral)	NI	NI
Badawy 2015 [13]	RCT	19/19	MC	5 (4.9)*^§^	6 (4.2)*^§^	11/8	14/5	NI	NI	NI	NI
El-Gohary 2012 [27]	Retrospective	11/18	SC	3.6 (3.47)*	2.42 (2.64)*	NI	NI	NI	NI	0	0
Canon 2007 [14]	Retrospective	29/20	SC	8.6 (4.9)	6.5 (4.2)	NI	NI	NI	NI	NI	NI

Statistical Analysis and Quantitative Data Synthesis

Quantitative data synthesis was performed using Review Manager (RevMan) (version 5.4.1; The Cochrane Collaboration, London, UK). Continuous outcomes including operative time, estimated blood loss, suturing duration, and length of hospitalization were analyzed using the mean difference (MD) with 95% confidence intervals (CIs) utilizing the inverse-variance method. Dichotomous outcomes, such as conversion rate, overall success rate, and complication incidence, were assessed using risk difference (RD) or risk ratio (RR) with 95% CIs, utilizing the Mantel-Haenszel method. Heterogeneity among studies was evaluated using the I² statistic. To ensure a conservative estimate and to rigorously account for potential between-study variance (clinical and methodological heterogeneity), a random-effects model was universally applied for all outcome syntheses, regardless of the I² value, because clinical heterogeneity across studies was anticipated a priori. The detailed breakdown of these statistical methodologies across all assessed outcomes can be found in Table 4.

**Table 4:** Statistical method for various outcomes.

Outcome	Data Type	Statistical Method	Analysis Model	Effect Measure
Operative Time	Continuous	Inverse Variance	Random Effects	Mean Difference (MD)
Hospital Stay	Continuous	Inverse Variance	Random Effects	Mean Difference (MD)
Complications	Dichotomous	Mantel-Haenszel	Random Effects	Risk Difference (RD)
Conversion	Dichotomous	Mantel-Haenszel	Random Effects	Risk Difference (RD)
Success Rate	Dichotomous	Mantel-Haenszel	Random Effects	Risk Ratio (RR)

Publication bias was assessed for all primary outcomes using visual inspection of funnel plot asymmetry and Egger's regression test, performed using the metafor package (version 5.0.1) in R Statistical Software (version 4.5.1; R Foundation for Statistical Computing, Vienna, Austria) [21]. Given the limited number of included studies (k=6), formal statistical tests for publication bias have restricted statistical power; therefore, visual inspection of funnel plot symmetry was considered the primary assessment, with Egger's test results interpreted with appropriate caution.

Sensitivity Analysis

To ensure the robustness of the findings, a sensitivity analysis was conducted by excluding studies with imputed missing data (e.g., standard deviations derived from ranges or p-values) to verify if the primary outcomes remained consistent without statistical imputation.

Meta-Regression and Non-linear Analysis

To investigate potential sources of heterogeneity and evaluate the influence of moderator variables on surgical outcomes, a random-effects meta-regression was performed using R Statistical Software (version 4.5.1; R Foundation for Statistical Computing, Vienna, Austria) with the metafor (version 5.0.1) [21] and meta (version 8.5.0) [22] packages. To analyze the non-linear relationship between patient age and conversion risk, a restricted cubic spline (RCS) regression was fitted with three knots placed at the 10th (1.92 years), 50th (4.44 years), and 90th (8.58 years) percentiles of the weighted mean study age distribution, following the recommendation of Harrell et al. for small datasets [23]. Given the small number of included studies (k = 6), this spline model was pre-specified as exploratory, and its results are interpreted as hypothesis-generating rather than confirmatory.

Univariable meta-regression models were constructed to assess the impact of: (1) publication year; (2) study design (retrospective vs. RCT); (3) center type (single-center vs. multicenter); and (4) country of origin. Furthermore, to explore potential non-linear relationships between patient age and surgical risks, an exploratory RCS regression was employed. Statistical significance for all regression analyses was defined as a p-value less than 0.05.

For dichotomous outcomes representing positive events (i.e., success rate), the effect measure was reported as an RR. Unlike adverse outcomes where a value below 1.0 suggests a benefit, an RR greater than 1.0 suggests a higher probability of success in the experimental group. To prevent ambiguity regarding the direction of the effect, the forest plot axes were explicitly labeled as 'Favours Retroperitoneal' and 'Favours Transperitoneal', ensuring that the visual representation aligns accurately with the clinical interpretation of the data.

For rare adverse events, such as open conversion and complications stratified by the Clavien-Dindo classification, the RD was utilized as the effect measure instead of the RR. By employing RD, these zero-event studies were successfully retained in the pooled analysis rather than being excluded, thereby ensuring a comprehensive assessment of safety profiles and providing a more precise estimate of absolute risk.

Results

Study Selection and Characteristics

The systematic database search identified 1,029 records (PubMed: n=107; Cochrane Library: n=32; Google Scholar: n=890). Following automated deduplication and removal of records marked ineligible by automated tools (n=26), 1,003 studies were screened by title and abstract, of which 393 were excluded. Of the remaining 610 studies assessed for full-text eligibility, 604 were excluded for the following reasons: wrong patient population (n=118), wrong study interventions (n=399), wrong outcomes design (n=4), incompatible language (n=7), and inaccessible full text (n=76). Six studies met all eligibility criteria and were included in both the systematic review and the quantitative meta-analysis (Figure 1).

The six included studies comprised one randomized controlled trial (Badawy et al. (2015) [13]) and five retrospective cohort studies (Canon (2007) [14], El-Gohary (2012) [27], Liu (2016) [26], Zhang (2019) [25], Marei (2025) [24]). The total study population was 1,010 patients (laparoscopic retroperitoneal (LR): n=588; laparoscopic transperitoneal (LT): n=422), drawn from centers across China, Egypt, the United Kingdom, and the United Arab Emirates, with publication dates ranging from 2007 to 2025. All six studies employed the Anderson-Hynes dismembered technique. Detailed baseline characteristics of all included studies are summarized in Table 3.

The total population collected from the inclusion studies was 1,010 patients, of whom 588 received laparoscopic retroperitoneal intervention and 422 received laparoscopic transperitoneal intervention. The population was collected from six inclusion studies [13,14,24-27]. All included studies utilized the Anderson-Hynes laparoscopic pyeloplasty technique. The summary of the meta-analysis can be seen in Table 5.

**Table 5:** Summary of meta-analysis. NE: Not Estimated (single study in subgroup). τ² reported in units of the effect measure squared (minutes² for operative time; days² for hospital stay; dimensionless for RD/RR).

Subgroup	No. of Studies	Sample Size		Heterogeneity				Overall Effect Size	95% CI of Overall Effect	P-Value
		LR	LT	I² (%)	P Value	τ²	Cochran’s Q (df)			
Operative time outcomes										
Retrospective	5	569	403	94	<0.00001	506.58	116.39 (4)	MD = 24.47	7.26 to 41.69	0.005
RCT	1	19	19	-	-	NE	NE	MD = -23.20	-40.06 to -6.34	0.007
Incidence of conversion to open surgery outcomes										
Retrospective	5	569	403	0	0.64	0.000	4.35 (4)	RD = 0.03	0.01 to 0.04	0.0005
RCT	1	19	19	-	-	NE	NE	RD = 0.00	-0.10 to 0.10	1.00
Success rate outcomes										
Retrospective	5	569	403	0	0.44	0.000	3.53 (4)	RR = 1.01	0.98 to 1.03	0.59
RCT	1	19	19	-	-	NE	NE	RR = 1.00	0.86 to 1.16	1.00
Duration of hospital stay outcomes										
Retrospective	5	569	403	92	<0.00001	90.4	52.2 (4)	MD = -0.03	-0.68 to 0.62	0.92
RCT	1	19	19	-	-	NE	NE	MD = -0.50	-0.84 to -0.16	0.004
Incidence of complication based on Clavien-Dindo grade outcomes										
Grade 1	5	137	111	0	0.77	0.0	1.63 (4)	RD = 0.00	-0.03 to 0.03	0.95
Grade 2	5	137	111	0	1.00	0.0	0.14 (4)	RD = -0.00	-0.03 to 0.03	0.94
Grade 3	5	137	111	0	0.87	0.0	0.83 (4)	RD = -0.03	-0.09 to 0.03	0.26

Quantitative Analysis Results Based on the Type of Outcome Analysis

Operative time outcomes: The analysis of operative duration across six studies (1,010 renal units) showed a non-significant trend favoring the transperitoneal approach (MD = 17.00 minutes; 95% CI = -2.04 to 36.04; P = 0.08; I²=96.6%; τ²=651.70 min²).

Subgroup analysis by study design revealed significant divergence (P < 0.001). Retrospective studies favored the transperitoneal approach (MD = 24.47 minutes; 95% CI = 7.26 to 41.69; P = 0.005), whereas the sole randomized controlled trial favored the retroperitoneal approach (MD = -23.20 minutes; 95% CI = -40.06 to -6.34; P = 0.007) [13].

**Figure 3:**
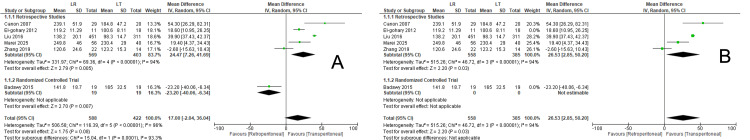
Meta-analysis of the operative time outcomes before sensitivity analysis (A) and after sensitivity analysis (B). Canon [14], El-Gohary [27], Liu [26], Marei [24], Zhang [25], Badawy [13]

Sensitivity analysis using the standardized mean difference (SMD = 0.79; 95% CI = -0.28 to 1.85; P = 0.15) confirmed the primary results. However, restricting analysis to four studies without imputed data [14,20-22] yielded a statistically significant advantage for the transperitoneal approach (MD = 26.53 minutes; 95% CI = 2.85 to 50.20; P = 0.03). Persistent heterogeneity (I² = 94%; P < 0.00001) remained, with individual study outcomes ranging from neutral [25,26] to strongly favoring the transperitoneal route. The forest plot of the operative time outcomes can be seen in Figure 3.

Incidence of Conversion to Open Surgery Outcomes

The analysis of conversion rates included data from all six studies (N = 1,010). Conversion from laparoscopy to open surgery was identified as a rare event, occurring in a total of 17 patients in the retroperitoneal group (2.9%) compared to only three patients in the transperitoneal group (0.7%). The RD was employed to incorporate studies with zero events in both arms, ensuring a more comprehensive safety synthesis.

The pooled analysis revealed a statistically significant difference favoring the transperitoneal approach. The pooled RD was 0.03 (95% CI = 0.01 to 0.04; P = 0.0006). This result suggests an absolute risk increase of 3% associated with the retroperitoneal approach.

**Figure 4:**
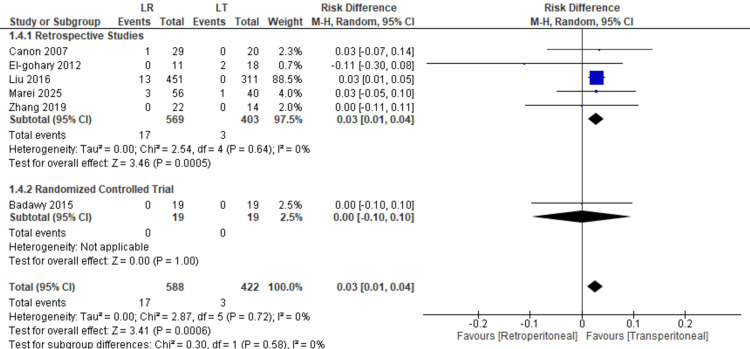
Meta-analysis of the conversion incidence outcomes. Canon [14], El-Gohary [27], Liu [26], Marei [24], Zhang [25], Badawy [13]

The analysis demonstrated perfect homogeneity (I² = 0%, τ²=0.000; P = 0.72), suggesting consistent safety outcomes across different centers. The finding was largely driven by the high-volume study by Liu et al. [26], which reported 13 conversions in the retroperitoneal group versus none in the transperitoneal group. The forest plot of the conversion incidence outcomes can be seen in Figure 4.

Success rate outcomes: For this outcome, surgical success is defined as a positive event; therefore, a RR greater than 1.0 suggests a trend favoring the transperitoneal approach. The cumulative analysis of surgical success rates incorporated data from all six studies, involving a total of 1,010 pediatric patients (422 in the transperitoneal group and 588 in the retroperitoneal group). Success was uniformly defined across studies as the resolution of symptoms and improvement in hydronephrosis on follow-up imaging. The pooled analysis using a Mantel-Haenszel random-effects model yielded a pooled RR of 1.01 (95% CI = 0.98 to 1.03; P = 0.60). This result suggests that the transperitoneal and retroperitoneal approaches are statistically equivalent in achieving successful resolution of ureteropelvic junction obstruction. The confidence interval is remarkably narrow (0.98-1.03), suggesting a high degree of precision in this finding. 

**Figure 5:**
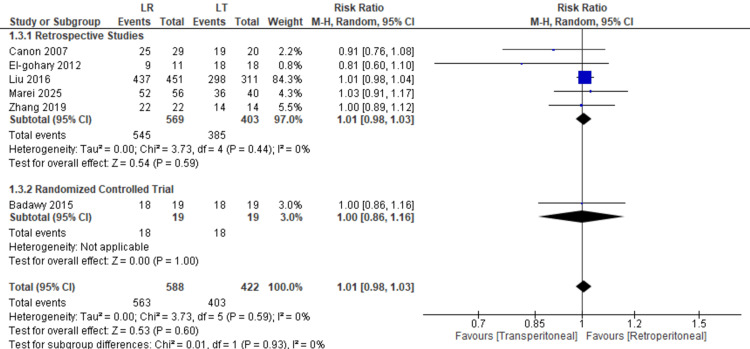
Meta-analysis of the success rate outcomes. Canon [14], El-Gohary [27], Liu [26], Marei [24], Zhang [25], Badawy [13]

Notably, this outcome demonstrated exceptional consistency across all included studies, with a heterogeneity (I²) value of 0%, τ²≈0 (P = 0.59). Unlike operative time or hospital stay, which varied significantly by institution, the success rate remained uniform regardless of the study design or sample size. Subgroup analysis confirmed this homogeneity, with the randomized controlled trial by Badawy et al. reporting an RR of 1.00 (95% CI = 0.86 to 1.16) and the retrospective studies collectively showing an RR of 1.01 (95% CI = 0.98 to 1.03) [13]. This confirms that while the choice of approach may impact the operative duration or length of stay, it does not compromise the ultimate clinical success of the pyeloplasty. The forest plot of the success rate outcomes can be seen in Figure 5.

Hospital stay outcomes: The analysis of postoperative hospital stay included data from all six studies, representing a total of 1,010 pediatric patients (422 in the transperitoneal group and 588 in the retroperitoneal group). The pooled analysis using a random-effects model demonstrated no statistically significant difference in the length of hospital stay between the two surgical approaches. The weighted MD was -0.14 days (95% CI = -0.65 to 0.37; P = 0.59), indicating that the duration of hospitalization is comparable regardless of the surgical technique employed.

Significant heterogeneity was observed across the included studies (I² = 90%; τ²=0.47 days², and P less than 0.00001). This high variability appears to stem from conflicting findings among the individual studies rather than a consistent trend. Specifically, the largest retrospective study [26] and the randomized controlled trial [13] both favored the retroperitoneal approach, reporting significantly shorter stays (MD = -1.00 days and -0.50 days, respectively) [13,26]. In contrast, earlier studies by Canon et al. [14] and El-Gohary et al. [27] favored the transperitoneal approach (MD = 0.70 days and 1.10 days, respectively) [14,23]. 

Other studies, such as those by Marei et al. and Zhang et al., reported nearly identical hospital stays for both groups (MD of approximately 0), further contributing to the overall conclusion of equivalence [24,25]. Consequently, while individual centers may observe shorter stays with a specific technique due to local protocols or surgeon expertise, the meta-analysis suggests that neither approach offers a universally superior advantage regarding hospital discharge timing.

To ensure that the finding of equivalence in hospital stay was not an artifact of statistical data conversion, a sensitivity analysis was performed by excluding studies that required the estimation of means from medians or the imputation of missing standard deviations. This rigorous exclusion limited the analysis to the two studies that explicitly reported raw mean and standard deviation values [14,25].

The sensitivity analysis corroborated the primary findings, demonstrating a pooled MD of 0.09 days (95% CI = -1.06 to 1.23; P = 0.88). This result confirms that there is no statistically significant difference in the length of hospital stay between the transperitoneal and retroperitoneal approaches when analyzing only direct raw data.

**Figure 6:**
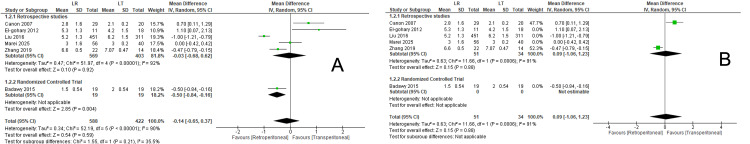
Meta-analysis of the hospital stay outcomes before sensitivity analysis (A) and after sensitivity analysis (B). Canon [14], El-Gohary [27], Liu [26], Marei [24], Zhang [25], Badawy [13]

However, heterogeneity remained notably high (I² = 91%; P = 0.0006), driven by the conflicting outcomes of the remaining studies. Canon et al. [14] reported a statistically significant reduction in hospital stay with the transperitoneal approach (MD = 0.70 days), whereas Zhang et al. [25] observed a trend favoring the retroperitoneal approach (MD = -0.47 days) [14,25]. The forest plot of the hospital stay outcomes can be seen in Figure 6.

The Incidence of Complications Based on Clavien-Dindo Grade Outcomes

To provide a granular assessment of safety, postoperative complications were stratified according to the Clavien-Dindo classification. This subgroup analysis incorporated data from five studies (N = 248), excluding the study by Liu et al. due to the absence of grade-specific reporting in their dataset [26]. The RD was utilized as the effect measure to allow for the inclusion of trials with zero events in both arms. The pooled analysis demonstrated no statistically significant difference in the incidence of complications across all severity grades (P = 0.68), with homogeneity (I² = 0%).

Grade I (minor deviations): Minor complications managed at the bedside (e.g., transient fever, minor wound issues) were observed in nine patients in the retroperitoneal group and seven in the transperitoneal group. The analysis showed an RD of 0.00 (95% CI = -0.03 to 0.03; P = 0.95), I²=0%; τ²=0, indicating an identical risk profile for minor adverse events.

Grade II (pharmacological intervention): Complications requiring pharmacological treatment, such as antibiotics for urinary tract infections or blood transfusion, were exceptionally rare, occurring in only one patient per group across the entire cohort. The pooled RD was -0.00 (95% CI = -0.03 to 0.03; P = 0.94), I²=0%; τ²=0, confirming that neither approach predisposes patients to infections or medical complications more than the other.

Grade III (surgical/radiological intervention): Severe complications requiring intervention under local or general anesthesia (e.g., stent migration, persistent leakage requiring percutaneous drainage) occurred in six patients in the retroperitoneal group and nine patients in the transperitoneal group. The analysis yielded an RD of -0.03 (95% CI = -0.09 to 0.03; P = 0.26), I²=0%; τ²=0. Although the transperitoneal group had a slightly higher raw number of interventions, the difference was not statistically significant.

**Figure 7:**
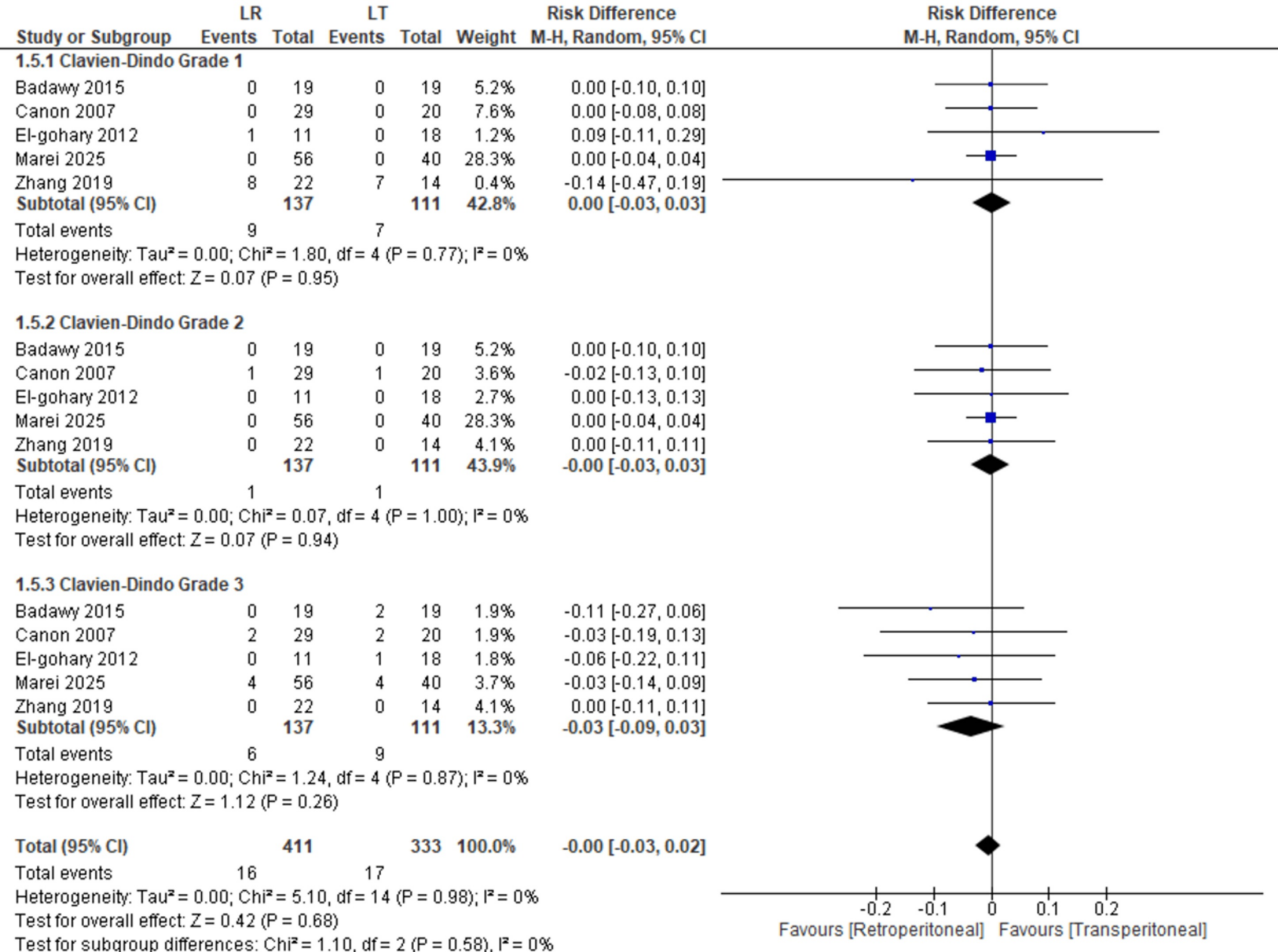
Meta-analysis of the complication incidence outcomes. This subgroup analysis incorporates data from five studies (N = 248) and excludes the study by Liu et al. [26] due to the absence of grade-specific reporting. Canon [14], El-Gohary [27], Marei [24], Zhang [25], Badawy [13]

Overall, the stratification confirms that both surgical approaches possess a comparable safety profile, with the vast majority of patients in both groups experiencing an uneventful recovery. The extensive overlap of confidence intervals crossing zero across all grades reinforces the conclusion that the choice of surgical approach does not influence the severity of postoperative complications. The forest plot of the complication incidence outcomes can be seen in Figure 7.

Meta-Regression

Influence of patient age on surgical outcomes: To investigate whether patient age serves as an effect modifier on the comparative efficacy and safety of transperitoneal versus retroperitoneal pyeloplasty, a random-effects meta-regression analysis was conducted. The results demonstrate that patient age is not a significant predictor of differential outcomes between the two surgical techniques (Table 6).

**Table 6:** Summary of meta-regression.

Outcome	Metric	Number of Studies (k)	Slope Coefficient (β)	95% CI	P-Value	Residual Heterogeneity (Ires2​)	R2 (Variance Explained)
Operative Time	MD	6	-2.2	-9.12 to 4.72	0.53	96.60%	0.00%
Conversion Rate	RD	6	-0.0008	-0.012 to 0.010	0.89	0.00%	0.00%
Hospital Stay	MD	6	-0.08	-0.25 to 0.09	0.36	89.70%	7.46%
Total Complications	RD	6	0.006	-0.012 to 0.024	0.52	0.00%	0.00%

For continuous variables, age did not significantly influence operative time (β = -2.20; 95% CI = -9.12 to 4.72; P = 0.53; I² = 96.6%; R² = 0.00%) or length of hospital stay (β = -0.08; 95% CI = -0.25 to 0.09; P = 0.36). Similarly, for categorical outcomes, patient age showed no significant correlation with success rate (β = 0.0009; P = 0.91; I² ~ 0%), risk of conversion to open surgery (β = -0.0008; P = 0.89; I² = 0.0%), or total complications (β = 0.006; P = 0.52; I² = 0.0%).

**Figure 8:**
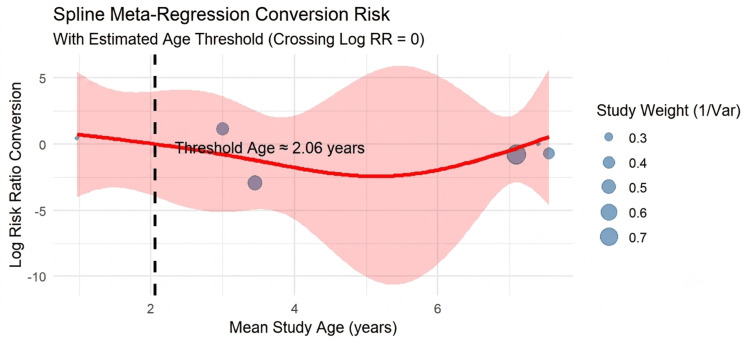
Bubble plot spline meta-regression. Bubble plot with restricted cubic spline regression illustrates the relationship between mean patient age and the risk of conversion (Log Risk Ratio). The vertical dashed line suggests an estimated inflection point at 2.06 years, suggesting a potential change in the risk profile associated with limited retroperitoneal working space in younger infants.

To further explore potential non-linear relationships that might be masked by standard linear regression, an RCS model was applied to the conversion rate data. While the linear model indicated no overall age effect, the spline analysis identified a potential inflection point at an estimated age of 2.06 years (Figure 8). Visual inspection of the spline curve suggests that the risk of conversion to open surgery exhibits greater variability and a potential upward trend in patients younger than two years. Beyond this threshold, the risk profile appears to stabilize, indicating that the technical feasibility of the retroperitoneal approach improves as the child's age and anatomical size increase. 

Methodological and Geographic Determinants

To elucidate the sources of the substantial heterogeneity observed in operative time and length of hospital stay (I² greater than 90%), and to assess the stability of clinical outcomes across different contextual factors, a random-effects meta-regression analysis was conducted. Four potential moderator variables were evaluated: publication year, study design, center type, and country of origin. The comprehensive results are summarized in Table 7.

**Table 7:** Comprehensive meta-regression analysis of moderator variables. *Statistically significant (P < 0.05). For "Study Design", the coefficient suggests that retrospective studies reported significantly larger time differences compared to RCTs. For "Country", the analysis identified significant heterogeneity in hospital stay protocols (R² ~ 70%), with UK and UAE reporting significantly shorter stays compared to China as reference. τ² Residual: Between-study variance remaining after accounting for the moderator. R² = proportion of total τ² explained by the moderator.

Moderator Variable	Outcome	Key Statistic	P-Value (QM​)	R2 (%)	τ² Residual	Interpretation
Patient Age	Operative Time	Slope: -2.20	0.53	0	790.01	No effect
	Hospital Stay	Slope: -0.08	0.36	7.46	0.5279	No effect
	Complications	Slope: 0.006	0.52	0	0	No effect
Publication Year	Operative Time	Slope: 1.64	0.44	0	735.88	No effect
	Hospital Stay	Slope: 0.05	0.32	0	0.4982	No effect
Study Design	Operative Time	Coeff: -47.73	0.035*	45.53	354.96	Retrospective studies exaggerate time diff.
(Ref: RCT)	Hospital Stay	Coeff: -0.45	0.27	15.6	0.5924	No significant bias
	Conversion Rate	Coeff: -0.02	0.58	0	0	No significant bias
Center Type	Operative Time	Coeff: -27.94	0.21	9.87	587.4	No effect
(Ref: Multicenter)	Hospital Stay	Coeff: -0.42	0.35	4.3	0.6246	No effect
Country	Operative Time	Omnibus Test	0.41	0	706.61	No geographic variation
(Ref: China)	Hospital Stay	Omnibus Test	0.013*	69.96	0.1417	Significant geographic variation
	Conversion Rate	Omnibus Test	0.5	0	0	Safety consistent globally

Meta-regression analysis revealed that publication year and center type (single vs. multicenter) were not significant predictors of any surgical outcome (P > 0.05).

However, significant moderators were identified for operative time and length of hospital stay. Study design significantly influenced operative time (β = -47.73; P = 0.035), explaining 45.53% of between-study heterogeneity. Retrospective studies showed a larger mean difference favoring the transperitoneal approach compared to the RCT. Country of origin was the primary determinant of hospital stay duration (P = 0.013), accounting for 69.96% of the heterogeneity (R² = 69.96%), with studies from the UK and UAE reporting shorter durations compared to the reference group (China).

Crucially, conversion and complication rates remained consistent across all moderator analyses (P > 0.05). These results indicate that both surgical approaches possess equivalent safety profiles regardless of study design, institutional protocols, or geographic location.

Certainty of Evidence

Grading of Recommendations, Assessment, Development, and Evaluation (GRADE) assessment: Evidence certainty was evaluated using the GRADE approach, with a baseline of "Low" due to the predominance of retrospective studies (five of six). Evidence for continuous outcomes (operative time and hospital stay) was graded as "Very Low" due to study design (risk of bias). However, meta-regression explained significant heterogeneity for operative time (R² = 45.5% attributed to study design) and hospital stay (R² ≈ 70% attributed to country of origin).

The success rate was graded as "Low" because no moderator variable significantly influenced this outcome (P > 0.05), supporting the consistency of both techniques. Safety outcomes (complications and conversion) were graded as "Very Low" due to imprecision and wide confidence intervals, although spline meta-regression identified a potential conversion risk threshold at 2.06 years. The comprehensive GRADE evidence profile is summarized in Table 8.

**Table 8:** Grade of evidence and recommendation. The certainty of evidence within the GRADE framework is symbolized using filled and open circles, stratified into four tiers: High (⊕⊕⊕⊕), indicating strong confidence that the true effect aligns closely with the estimate; Moderate (⊕⊕⊕◯), reflecting reasonable confidence where the true effect is likely near the estimate but could differ substantially; Low (⊕⊕◯◯), showing restricted confidence as the actual effect might vary significantly; and Very Low (⊕◯◯◯), denoting minimal confidence with a high probability that the true effect differs substantially from the pooled estimate.

Outcome	Certainty (GRADE)	Impact of Meta-Regression Analysis
Operative Time	⨁◯◯◯ Very Low	Explained Inconsistency: Meta-regression revealed that 45% of heterogeneity is driven by Study Design bias. Retrospective studies exaggerate the time difference compared to RCTs.
Hospital Stay	⨁◯◯◯ Very Low	Explained Inconsistency: Meta-regression revealed that 70% of heterogeneity is driven by Country protocols. The variation reflects healthcare systems, not surgical failure.
Success Rate	⨁⨁◯◯ Low	Confirmed Consistency: Meta-regression confirmed that success rates are stable across all moderators (Age, Year, Center). Downgraded solely due to Risk of Bias (Retrospective data).
Conversion Rate	⨁◯◯◯ Very Low	Identified Threshold: Spline regression identified a potential risk inflection point at age < 2.06 years, suggesting lower certainty for infants due to anatomical constraints.

Publication Bias Assessment

**Figure 9:**
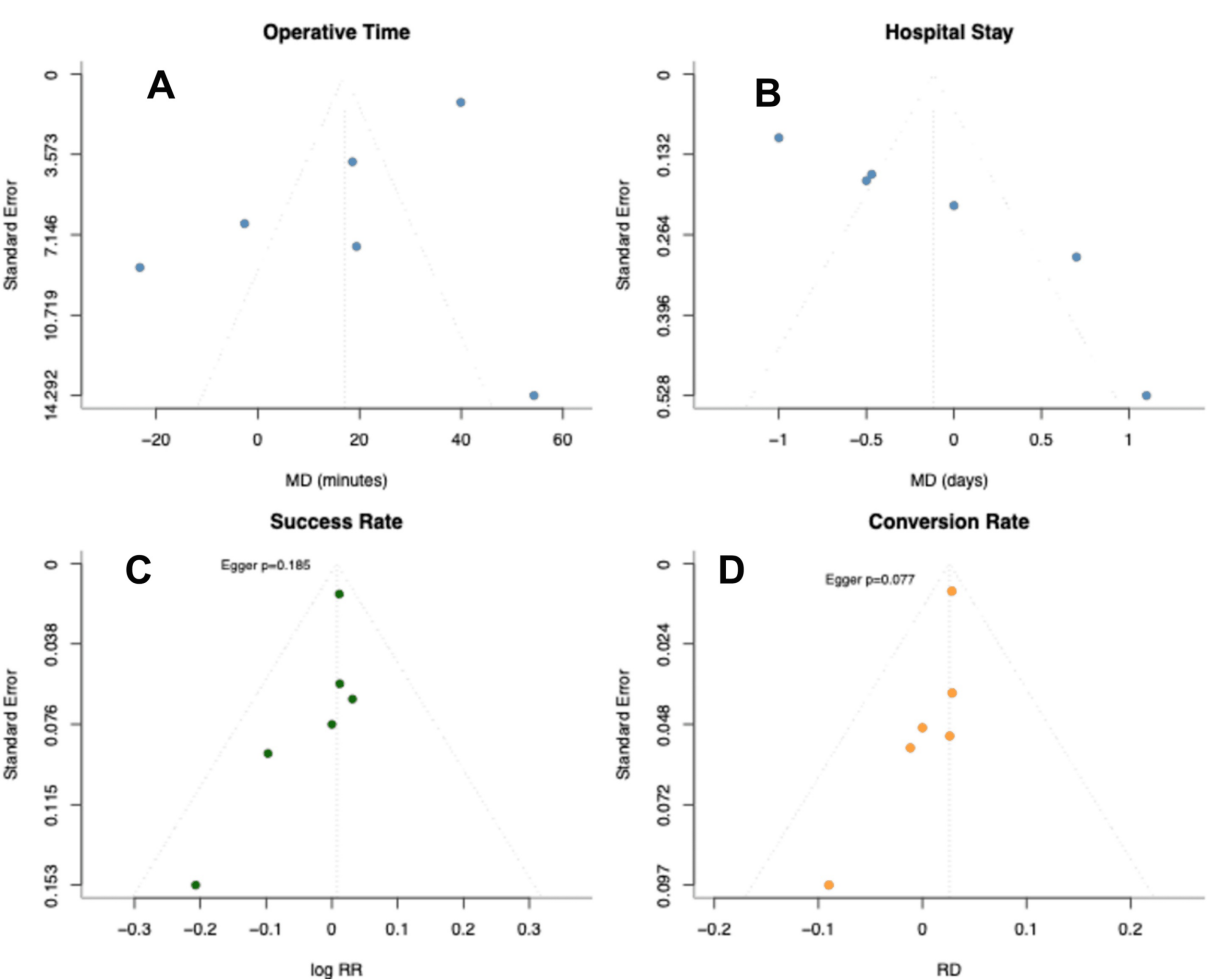
Funnel plots used to assess potential publication bias for each meta-analysis outcome. Funnel plots for all primary meta-analysis outcomes. Studies distributed approximately symmetrically for operative time, success rate, and conversion rate. For hospital stay, visual asymmetry was noted and confirmed by Egger's test (p=0.003); this likely reflects genuine geographic heterogeneity rather than publication bias, as discussed in the text. RR: Risk ratio; RD: risk difference

Funnel plots and Egger’s regression tests were utilized to assess publication bias (Figure 9; Table 9). Visual symmetry and non-significant Egger’s tests indicated no substantial publication bias for operative time (z = -2.242; P = 0.088), surgical success rate (z = -1.600; P = 0.185), and conversion rate (z = -2.368; P = 0.077).

**Table 9:** Results of Egger's regression test for publication bias across all analyzed outcomes.

Outcome	Egger_z	Egger_p	Interpretation	Caveat
Operative Time	-2.242	0.0884	Symmetric (p>=0.05)	k=6 limits power of Egger's test
Hospital Stay	6.285	0.0033	Asymmetric (p<0.05)*	k=6 limits power of Egger's test
Success Rate	-1.6	0.1849	Symmetric (p>=0.05)	k=6 limits power of Egger's test
Conversion Rate	-2.368	0.077	Symmetric (p>=0.05)	k=6 limits power of Egger's test

For hospital stay duration, Egger’s test indicated significant asymmetry (z = 6.285; P = 0.003). However, given the limited number of studies (k = 6), this result should be interpreted with caution. The observed asymmetry likely reflects the genuine clinical and geographic heterogeneity previously identified, rather than publication bias.

Discussion

This systematic review and meta-analysis provide a comprehensive evaluation of the comparative efficacy and safety of transperitoneal versus retroperitoneal laparoscopic pyeloplasty in the pediatric population [6,7]. The primary finding of our study is that both surgical approaches demonstrate comparable therapeutic success rates and safety profiles [7]. Specifically, no statistically significant differences were observed in the overall rates of postoperative complications or surgical success between the two techniques [5,6]. These results suggest that the choice of surgical approach can be flexibly adapted based on surgeon expertise and patient-specific anatomical considerations rather than being dictated by a definite superiority of one technique over the other [5,7].

Divergence Between Study Designs: Operative Time and Conversion

A critical observation in our analysis is the divergence of outcomes between study designs, particularly regarding operative time and conversion rates. While the overall pooled estimate showed high heterogeneity, with an I² value greater than 90%, our meta-regression identified study design as a significant moderator (P = 0.035). Interestingly, the RCT subgroup suggested a trend toward shorter operative times with the retroperitoneal approach, whereas the retrospective observational subgroup favored the transperitoneal approach, associating it with significantly shorter durations and, notably, a lower risk of conversion to open surgery [13].

This discrepancy mandates a cautious interpretation. The favorable outcomes for the transperitoneal approach in retrospective studies may reflect a selection bias and the learning curve phenomenon inherent in real-world practice [7]. In observational settings, surgeons may prefer the transperitoneal route, which offers a larger working space and familiar anatomical landmarks, resulting in fewer technical difficulties and conversions [13]. Conversely, the retroperitoneal approach might be technically more demanding for less experienced surgeons or selectively utilized for complex cases in non-randomized cohorts, thereby inflating operative times and conversion rates. Thus, while the retroperitoneal approach demonstrates high efficiency in expert hands (as seen in RCTs) [13], the transperitoneal route appears to offer a more consistent safety profile with lower conversion risks in broader clinical practice [5,7]. The choice of surgical approach (transperitoneal vs. retroperitoneal) may depend more on surgeon experience and specific patient characteristics than on a universal time advantage.

Geographic Variation in Hospitalization

Regarding the length of hospital stay, our meta-regression revealed that the substantial heterogeneity observed was largely driven by geographic location (P = 0.013; R² value of approximately 70%). Studies conducted in the United Kingdom and the United Arab Emirates reported significantly shorter hospital stays compared to those from China, regardless of the surgical technique used [24-27]. This variation likely reflects systemic differences in healthcare protocols, such as the implementation of Enhanced Recovery After Surgery (ERAS) pathways in Western and Middle Eastern centers [29], versus more conservative observational protocols in other regions. This suggests that hospital discharge timing is less dependent on the surgical approach and more likely influenced by non-clinical factors, such as institutional discharge protocols, healthcare systems, and patient age demographics. Importantly, despite these differences in discharge timing, the complication and readmission rates remained comparable across all studies, implying that early discharge following either laparoscopic approach is feasible and safe [29].

Impact of Patient Age and Anatomical Constraints

A key strength of this study is the granular assessment of patient age as a moderator. Our linear meta-regression demonstrated that patient age does not significantly influence the primary surgical outcomes (P greater than 0.05), suggesting that laparoscopic pyeloplasty is a robust option across the pediatric age spectrum [30]. However, our exploratory non-linear spline analysis identified a potential age threshold at approximately 2.06 years regarding the risk of conversion. This finding is biologically plausible, as the retroperitoneal working space in infants and toddlers is anatomically restricted compared to older children [26]. While the overall safety profile remains high, this finding suggests that the working space constraint in children under two years of age may necessitate a higher level of surgical precision and experience to avoid conversion, particularly when employing the retroperitoneal approach [8].

Publication Bias Considerations

Funnel plot asymmetry was not detected for operative time, success rate, or conversion rate. For hospital stay, Egger's test indicated statistically significant asymmetry (p=0.003); however, this finding should be interpreted with caution given the limited number of included studies (k=6), which substantially reduces the power and specificity of Egger's test [31]. As discussed under geographic variation, the observed asymmetry is most plausibly attributable to the documented country-level differences in discharge protocols (R²=70%), which create systematic between-study differences that manifest as funnel asymmetry, rather than selective non-publication of studies reporting shorter hospital stays. This interpretation is supported by the consistency of complication and readmission rates across all included centers, suggesting that differences in discharge timing reflect institutional policies rather than differential study reporting.

Limitations

Several limitations of this study must be acknowledged. First, the certainty of the evidence, assessed using the GRADE framework, ranges from low to very low, primarily due to the predominance of retrospective studies and the inherent risk of selection bias. Second, although we employed rigorous search strategies, the inclusion of grey literature from Google Scholar without strictly predefined screening thresholds may have introduced a potential selection bias. Third, the RCS analysis is constrained by the small number of included studies (k=6), which limits the precision and stability of the estimated inflection point (~2.06 years) and precludes definitive conclusions regarding the age threshold for conversion risk. Finally, the lack of long-term follow-up data (greater than five years) in most studies precludes a definitive comparison of late recurrence rates between the two techniques.

## Conclusions

In conclusion, transperitoneal laparoscopic pyeloplasty and retroperitoneal laparoscopic pyeloplasty are comparable in terms of success rates, safety, and overall complication risks for pediatric patients. However, our analysis reveals a nuanced distinction based on study design: while randomized data suggest potential efficiency benefits for the retroperitoneal approach, observational evidence highlights the transperitoneal approach as having a significantly lower risk of conversion and shorter operative times in retrospective settings. Neither approach demonstrated clear clinical superiority; hence, the surgical choice should be individualized. The transperitoneal approach may remain the preferred option for surgeons seeking a wider working space and a lower risk of conversion, particularly during the initial learning phase. Conversely, the retroperitoneal approach represents a valuable alternative that offers excellent efficiency, provided the surgeon has overcome the learning curve. Clinical decision-making should be tailored to the surgeon’s experience level and the patient’s specific anatomical characteristics, with particular caution warranted for infants under two years of age due to potential anatomical space constraints.
